# The prevalence of mild cognitive impairment in Gulf War veterans: a follow-up study

**DOI:** 10.3389/fnins.2023.1301066

**Published:** 2024-01-22

**Authors:** Linda L. Chao, Kimberly Sullivan, Maxine H. Krengel, Ronald J. Killiany, Lea Steele, Nancy G. Klimas, Bang-Bong Koo

**Affiliations:** ^1^Department of Radiology and Biomedical Imaging, University of California, San Francisco, San Francisco, CA, United States; ^2^Department of Psychiatry and Behavioral Sciences, University of California, San Francisco, San Francisco, CA, United States; ^3^San Francisco Veterans Affairs Health Care System, San Francisco, CA, United States; ^4^Department of Environmental Health, Boston University School of Public Health, Boston, MA, United States; ^5^Department of Neurology, Boston University School of Medicine, Boston, MA, United States; ^6^Department of Anatomy and Neurobiology, Boston University School of Medicine, Boston, MA, United States; ^7^Department of Psychiatry and Behavioral Sciences, Baylor College of Medicine, Houston, TX, United States; ^8^Dr. Kiran C. Patel College of Osteopathic Medicine, Institute for Neuro-Immune Medicine, Nova Southeastern University, Fort Lauderdale, FL, United States; ^9^Geriatric Research Education and Clinical Center (GRECC), Miami VA Medical Center, Miami, FL, United States

**Keywords:** cognition, mild cognitive impairment, Gulf War, neuropsychology, hippocampus, neuroimaging

## Abstract

**Introduction:**

Gulf War Illness (GWI), also called Chronic Multisymptom Illness (CMI), is a multi-faceted condition that plagues an estimated 250,000 Gulf War (GW) veterans. Symptoms of GWI/CMI include fatigue, pain, and cognitive dysfunction. We previously reported that 12% of a convenience sample of middle aged (median age 52 years) GW veterans met criteria for mild cognitive impairment (MCI), a clinical syndrome most prevalent in older adults (e.g., ≥70 years). The current study sought to replicate and extend this finding.

**Methods:**

We used the actuarial neuropsychological criteria and the Montreal Cognitive Assessment (MoCA) to assess the cognitive status of 952 GW veterans. We also examined regional brain volumes in a subset of GW veterans (*n* = 368) who had three Tesla magnetic resonance images (MRIs).

**Results:**

We replicated our previous finding of a greater than 10% rate of MCI in four additional cohorts of GW veterans. In the combined sample of 952 GW veterans (median age 51 years at time of cognitive testing), 17% met criteria for MCI. Veterans classified as MCI were more likely to have CMI, history of depression, and prolonged (≥31 days) deployment-related exposures to smoke from oil well fires and chemical nerve agents compared to veterans with unimpaired and intermediate cognitive status. We also replicated our previous finding of hippocampal atrophy in veterans with MCI, and found significant group differences in lateral ventricle volumes.

**Discussion:**

Because MCI increases the risk for late-life dementia and impacts quality of life, it may be prudent to counsel GW veterans with cognitive dysfunction, CMI, history of depression, and high levels of exposures to deployment-related toxicants to adopt lifestyle habits that have been associated with lowering dementia risk. With the Food and Drug Administration’s recent approval of and the VA’s decision to cover the cost for anti-amyloid β (Aβ) therapies, a logical next step for this research is to determine if GW veterans with MCI have elevated Aβ in their brains.

## 1 Introduction

An estimated 250,000 veterans of the 1991 Gulf War (GW) suffer from a chronic, multisymptomatic condition (e.g., persistent fatigue, musculoskeletal pain, sleep, gastrointestinal, and respiratory problems, skin rashes, and cognitive dysfunction) that has been linked to military service in the 1991 Persian Gulf War (Steele, 2000; Institute Of Medicine [IOM], 2010; White et al., 2016). Cognitive dysfunction is not only a common symptom among veterans suffering from this chronic multi-faceted illness (Smith et al., 2013; Janulewicz et al., 2017; Jeffrey et al., 2019), but it is also a defining symptom category for the two most widely used definitions for classifying this chronic condition in GW veterans, the Kansas Gulf War Illness (GWI) case definition (Steele, 2000) and the Centers for Disease Control and Prevention Chronic Multisymptom Illness (CDC CMI) case definition (Fukuda et al., 1998).

In the past, it was satisfactory for clinicians to distinguish dementia from the cognitive decline typically accompanies normal aging. However, with recent advances in biomarker imaging and plasma-based assays and the advent of neuroprotective therapies, neurologists can now make finer-grained distinctions about where on the clinical spectrum from preclinical cognitive decline to dementia a patient falls (Jack et al., 2018). Mild cognitive impairment (MCI) is central to this diagnostic scheme (Petersen, 2004). Regarded as a transitional stage between healthy aging and early dementia, patients with MCI exhibit cognitive impairment greater than expected for their age, yet not severe enough to meet criteria for dementia (Tangalos and Petersen, 2018). Because the incidence of MCI increases with age (Ganguli et al., 2013), most research studies on MCI have focused on adults over 60 years old. However, a recent meta-analysis/systematic review estimated that 7.6% of adults between 55 and 59 years have MCI (Lu et al., 2021). Notably, that estimate is lower than the 12% that we recently reported in a convenience sample of 202 middle-aged (median age 52 years old) GW veterans (Chao, 2020).

Clinically, MCI has been viewed as a “window” in which it may be possible to intervene and delay progression to dementias such as Alzheimer’s disease (AD) (Anderson, 2019). Therefore, the first aim of this study is to replicate our previous finding of a higher-than-expected rate of MCI in additional cohorts of GW veterans. Hippocampal atrophy is a prominent feature of AD and MCI (Killiany et al., 2000; Apostolova et al., 2006; Frisoni et al., 2010) that has been used as a preclinical and disease progression marker of AD (Tapiola et al., 2008; Tanpitukpongse et al., 2017). Thus, a second aim of this study was to replicate our previous finding of hippocampal atrophy in the GW veterans with MCI compared to cognitively unimpaired GW veterans (Chao, 2020). Ventriculomegaly, enlargement of the ventricles, is commonly observed in patients with MCI and AD (Thompson et al., 2004; Nestor et al., 2008) and often co-occurs with hippocampal atrophy (Apostolova et al., 2012). Wu et al. (2021) recently described ventricular enlargement and hippocampal atrophy in an animal model of GWI. In exploratory analyses, we examined the volumes of the lateral ventricles in veterans with and without MCI. Finally, research suggests that posttraumatic stress disorder (PTSD), depression, and environmental exposures to neurotoxicants may be associated with cognitive dysfunction (Lebowitz et al., 1997; Golier et al., 2002; Charney et al., 2003; Yehuda et al., 2006; Clouston et al., 2019). Because these conditions have been documented in GW veterans (Labbate and Snow, 1992; Sutker et al., 1995; Research Advisory Committee on Gulf War veterans’ Illnesses [RAC-GWVI], 2008; Research Advisory Committee on Gulf War Veterans’ Illnesses [RAC-GWVI], 2014; Blore et al., 2015), we also explored the relationship between the veterans’ cognitive status and their history of PTSD, depression, and deployment-related exposures.

## 2 Materials and methods

### 2.1 Participants

We analyzed data from five cohorts of GW veterans: The San Francisco (SF) 3T cohort consisted of the 202 GW veterans described our original 2020 report (Chao, 2020). The Gulf War Illness Consortium (GWIC) cohort consisted of 262 GW veterans from the Boston University-based GWIC study (Steele et al., 2021). The SF 4T cohort consisted of 170 GW veterans who participated in a 4 Tesla (T) magnetic resonance image (MRI) research study based at the San Francisco Veterans Affairs Health Care System (SFVAHCS) (Chao et al., 2011). The SF 1.5T cohort consisted of 241 GW veterans who participated in a 1.5T MRI research study at the SFVAHCS (Chao et al., 2010; Weiner et al., 2011). The San Francisco Montreal Cognitive Assessment (SF MoCA) cohort consisted of 77 GWVs who participated in a remote study that used the MoCA (Nasreddine et al., 2005) to evaluate cognitive function. All the veterans study participants had been deployed to the Persian Gulf War between August 1990 and July 1991. Table 1 summarizes the demographic characteristics of each cohort.

**TABLE 1:** Demographic and clinical characteristics by cohort.

	SF 1.5T	SF 4T	SF 3T	GWIC	SF MoCA
Years of study	2002–2007	2005–2010	2014–2018	2015–2020	2020–2023
*N*	241	170	202	262	77
**Age, years**					
Mean (SD)	45.3 (9.9)	49.3 (8.3)	54.2 (7.8)	52.2 (5.9)	60.2 (7.5)
Range	31–71	34–70	41–77	42–79	50–81
Median	44	49	53	51	59
**Education, years**					
Mean (SD)	14.4 (1.9)	15.2 (2.2)	15.6 (2.3)	14.7 (2.1)	14.9 (2.2)
Range	10–20	12–20	12–20	12–21	12–20
Median	14	15	16	14	14
Male, *N* (%)	208 (86.3%)	150 (88.2%)	165 (81.7%)	219 (83.6%)	73 (94.8%)
Hispanic ethnicity, *N* (%)	23 (9.5%)	34 (20%)	20 (10%)	21 (8.2%)	6 (8%)
**Race, N (%)**					
Black	40 (16.6%)	17 (10%)	18 (8.9%)	34 (13%)	3 (4.1%)
White	154 (63.9%)^c^	102 (60.0%)^c^	148 (73.3%)	208 (79.4%)	67 (91.8%)
Other^a^	47 (19.5%)^d^	51 (30.0%)^d^	36 (17.8%)	20 (7.6%)	3 (4.1%)
CDC CMI,^b^ *N* (%)	158 (65.6%)	107 (62.9%)	152 (75.2%)	235 (90%)	77 (100%)
Kansas GWI, *N* (%)	–	–	91 (45%)	218 (83.5%)	0 (0.0%)
Kansas GWI exc. *N* (%)	–	–	47 (23.3%)	–	48 (62.3%)
Khamisiyah exposed *N* (%)	36 (14.9%)	63 (37.1%)	88 (43.6%)	–	–
PTSD, *N* (%)	70 (29.5%)^e^	41 (24.1%)^e^	72 (35.6%)^e^	134 (53.4%)^f^	24 (31.2%)^f^
Depression, *N* (%)	105 (43.8%)^g^	62 (36.5%)^g^	22 (10.9%)^g^	128 (48.9%)^f^	18 (23.4%)^f^

^*a*^Includes Asians, Native Americans/Hawaiians, Pacific Islanders, multi- and mixed races.

^*b*^Centers for Disease Control and Prevention Chronic Multisymptom Illness, includes fatigue, joint + muscle pain, difficulty sleeping, remembering, concentrating.

^*c*^Does not include Hispanic Whites.

^*d*^Includes Hispanic/Latinos.

^*e*^Determined by Clinician Administered PTSD Scale (CAPS) (lifetime PTSD).

^*f*^Detetermined from self-report.

^*g*^Detetermined by Structured Clinical Interview for DSM IVI (lifetime Major Depressive Disorder).

### 2.2 Neuropsychological battery

To ascertain actuarial MCI status in the GW veterans from the SF 1.5T, SF 4T, SF 3T, and GWIC cohorts, we used raw neuropsychological test scores to assess three cognitive domains (verbal memory, executive functioning, and attention). Two separate measures were used to assess each cognitive domain. Delayed free recall and total recall scores from trials 1 to 5 from the California Verbal Learning Test (CVLT)-II (Delis et al., 2000) were used to assess verbal memory. Time to compete Trial-Making Test, Part B (TMT-B) (Reitan and Wolfson, 1985), time to complete the Delis-Kaplan Executive Function System (D-KEFS) (Delis et al., 2001) color word interference, switching condition, and/or raw scores on the Short Category test (Wetzel and Boll, 1987) were used to assess executive function. Time to complete TMT-A (Reitan and Wolfson, 1985), Wechsler Adult Intelligence Scale (WAIS) III (Wechsler, 1997) Digit Span score, and/or the *d*′ score from the Conners Continuous Performance Test version 3 (CPT-3) (Conners, 2014) were used to assess the attention domain. Table 2 summarizes the neuropsychological measures that were used for each domain in the different cohorts. Table 2 also lists the neuropsychological measures and cognitive domains that were used to ascertain neuropsychological actuarial criteria cohort in our 2020 study (Chao, 2020).

**TABLE 2:** Neuropsychological test measures and cognitive domains used to determine actuarial MCI status.

	Cognitive domains				
Cohort	Verbal memory	Executive function	Attention	Language	Visuospatial function
Original study (Chao, 2020)	• CVLT-2 trials 1–5	• TMT-B	• TMT-A	• Boston Naming Test	• WAIS III Block Design
	• CVLT-2 LDFR	• D-KEFS color/word switching	• WAIS-III Digit Span		
Re-analysis of SF 2020 cohort	• CVLT-2 trials 1–5	• TMT-B	• TMT-A		
	• CVLT-2 LDFR	• D-KEFS color/word switching	• Conners CPT *d*′		
GWIC cohort	• CVLT-2 trials 1–5	• TMT-B	• TMT-A		
	• CVLT-2 LDFR	• DKEFS color/word switching	• Conners CPT *d*′		
SF 4T cohort	• CVLT-2 trials 1–5	• TMT-B	• TMT-A		
	• CVLT-2 LDFR	• Halstead-Reitan category test	• WAIS-III Digit Span		
SF 1.5T cohort	• CVLT-2 trials 1–5	• TMT-B	• TMT-A		
	• CVLT-2 LDFR	• Halstead-Reitan category test	• WAIS-III Digit Span		

GWIC, Gulf War Illness Consortium; CVLT, California Verbal Learning Test; LDFR, Long-Delay Free Recall; D-KEFS, Delis-Kaplan Executive Function System; TMT, Trail-Making Test; WAIS, Weschler Adult Intelligence Scale.

### 2.3 Actuarial neuropsychological criteria

In the original iteration of the actuarial MCI criteria, Jak et al. (2009a) employed a comprehensive neuropsychological battery that assessed five cognitive domains (memory, attention, language, visuospatial functioning, and executive functioning) with three measures per domain. Later, Bondi et al. (2014) modified the comprehensive actuarial MCI criteria to include one impaired score across three domains, or a Functional Assessment Questionnaire (FAQ) score >9. In our 2020 study (Chao, 2020), we used the same five cognitive domains described by Jak et al. (2009a). However, because we only had one measure in the visuospatial and language domains, we modified the actuarial criteria to include two measures for the verbal memory, executive function, and attention domains and one measure in the language and visuospatial function domain (see Table 2).

For this study, we operationalized the actuarial MCI criteria as Bondi et al. (2014) with the Alzheimer’s Disease Neuroimaging Initiative (ADNI) cohort by utilizing two measures in three cognitive domains: verbal memory, executive attention, and attention. Raw neuropsychological scores were transformed into demographically adjusted *z*-scores (accounting for age, sex, education, and race) for each participant on each neuropsychological measure using the Revised Heaton Norms (Heaton et al., 2004), the Halstead-Reitan (Reitan and Wolfson, 1985), D-KEFS (Delis et al., 2001), and Conners CPT-3 (Conners, 2014) manuals. A score was considered impaired if it fell more than 1 SD below the corresponding demographically adjusted normative mean (i.e., *Z* < −1.0). Veterans with two impaired scores in one domain or two impaired scored in two or more domains were classified as MCI. Veterans with one impaired score in two or more domains were classified as intermediate. Veterans with no impaired scores in any cognitive domain were classified as cognitively unimpaired.

### 2.4 Montreal cognitive assessment

We administered the MoCA, a test with excellent test-retest reliability and internal consistency designed to identify multidomain MCI (Nasreddine et al., 2005), remotely to 77 GW veterans between January 2020 and May 2023. A validated 30-point screening tool for MCI (Nasreddine et al., 2005), the MoCA assesses eight cognitive domains including short-term memory, visuospatial abilities, executive function, attention, concentration, working memory, language, and orientation to space and time. In a manner consistent with the *National Institute on Aging–Alzheimer’s Association’s* definition for MCI (Albert et al., 2011), we used a MoCA cut-off of 23 to identify veterans with MCI in the SF MoCA cohort.

### 2.5 Brain magnetic resonance imaging

We examined 3T structural MRIs of GW veterans from the SF 3T (*n* = 197), GWIC cohort (*n* = 155), and the MoCA (*n* = 16) cohorts. Because the SF MoCA cohort was assessed remotely, we only have neuroimaging data in small subset of participants from this cohort. The imaging parameters for these studies have been described previously (Chao, 2020; Steele et al., 2021).

### 2.6 Image processing

Hippocampal and lateral ventricle volumes were estimated with FreeSurfer version 7.1. We also used the total intracranial volume estimate (eTIV) from FreeSurfer to account for inter-individual variations in brain volume due to head size differences. Previous studies have reported subcortical (i.e., brainstem, thalamus, ventral diencephalon, and cerebellum) atrophy in GWI (Christova et al., 2017; Zhang et al., 2020). Therefore, in exploratory analyses, we also examined the effects of CMI and GWI status on the volumes of these subcortical regions of interest.

### 2.7 Chronic multisymptom illness criteria

The CDC CMI (Fukuda et al., 1998) case status was operationalized as the presence of persistent symptoms over 6 months in two out of three domains: fatigue (lack of energy/overly tired), musculoskeletal (joint and/or muscle pain), and cognitive/mood (difficulty remembering, difficulty concentrating, and trouble sleeping). We used self-report health questionnaires to ascertain CDC CMI case status.

### 2.8 Kansas Gulf War illness criteria

The Kansas GWI case status requires veterans to have moderately severe or multiple mild chronic symptoms in at least three of six categories: fatigue/sleep problems, pain, neurological, cognitive and mood symptoms, respiratory complaints, gastrointestinal problems or skin symptoms (Steele, 2000). The Kansas GWI case criteria also excludes veterans who have psychiatric conditions that may interfere with the accurate reporting of symptoms and/or medical conditions that might predict similar symptoms as GWI. The Kansas Gulf War Military History and Health Questionnaire (Steele, 2000) was used to ascertain Kansas GWI case status in the GWIC, SF 3T, and SF MoCA cohorts. Because this questionnaire was not part of the earlier SFVAHCS protocols, we do not have information about Kansas GWI case status for veterans from the SF 1.5T and SF 4T cohorts.

### 2.9 Predicted Khamisiyah exposure status

In March 1991, U.S. troops detonated a munitions storage pit at Khamisiyah, Iraq that was later found to contain stockpiles of chemical nerve agents sarin and cyclosarin (Department of Defense [DoD], 2002). The plume that resulted from this demolition operation exposed potentially more than 100,000 U.S. troops to low levels of sarin and cyclosarin. We requested information about Khamisiyah exposure status of veterans from SF 1.5T, SF 4T, and SF 3T cohorts from the Deputy Assistant Secretary of Defense for Force Health Protection and Readiness as previously described (Chao et al., 2010). We did not have information about the Khamisiyah exposure for veterans from the GWIC or SF MoCA cohorts.

### 2.10 Gulf War-related exposures

In addition to asking questions about health symptoms, the Kansas Gulf War Military History and Health Questionnaire also queried veterans about deployment-related exposures to chemical weapons, pesticides, and anti-nerve gas pills during their 1991 deployment (Steele, 2000). Exposure to chemical warfare agents was determined by self-reports of hearing chemical alarms sound. Particulate matter exposure was determined by self-reports of seeing smoke from oil well fires. Pesticide exposure was determined by self-reports of using cream or spay pesticide directly on the skin and seeing living area sprayed or fogged with pesticides. Anti-nerve gas pill exposure was determined by self-reports taking pyridostigmine bromide (PB) pills (Sullivan et al., 2018). The Kansas questionnaire asked veterans about the duration of the exposures (i.e., none, 1–6 days, 7–30 days, ≥31 days) to approximate the dose of the exposures. Because the Kansas questionnaire was not administered as part of the earlier SF studies, we only have information about deployment-related exposures for veterans from the GWIC, SF 3T, and SF MoCA cohorts.

### 2.11 PTSD

Posttraumatic stress disorder was assessed with the Clinician Administered PTSD Scale (CAPS) (Blake et al., 1995) in the SF 1.5T, SF 4T, and SF 3T cohorts. In the GWIC and SF MoCA cohorts, information about PTSD was obtained through self-reports of psychiatric history. In an attempt to reconcile the different methods used to ascertain PTSD status, we used lifetime PTSD instead of current PTSD status for the veterans who were assessed with the CAPS.

### 2.12 Depression

Depression was assessed with the Structured Clinical Interview for DSM IV (SCID) (First et al., 1995) in the SF 1.5T, SF 4T, and SF 3T cohorts. In the GWIC and SF MoCA cohorts, information about depression was obtained through self-reports of psychiatric history. As with PTSD, we used lifetime rather than current Major Depressive Disorder to quantify depression status in the veterans who were assessed with the SCID.

### 2.13 Data analysis

Statistical analyses were conducted with the Statistical Package for the Social Sciences (SPSS) Version 29. Mean values of continuous variables were compared using analysis of variance (ANOVA) or analysis of covariance (ANCOVA) with Tukey honest significant difference (HDS) *post-hoc* tests. Proportional comparisons were assessed with Chi-square tests. Multiple linear regression was used to examine the ability of demographic (e.g., age, sex, and years of education) and clinical variables (e.g., PTSD, depression, and CMI case status) to predict cognitive status (i.e., unimpaired, intermediate, and MCI).

The analyses of regional brain volume were carried out in stages. Because we sought to replicate our previous finding of smaller hippocampal volumes in veterans with MCI, we initially excluded the SF 3T cohort from the one-way analysis covariance (ANCOVA) of hippocampal volume. Age, male sex, education, PTSD diagnosis, and eTIV were included as covariates in the analysis. We also included Kansas GWI exclusionary status as a covariate to account for current health status.

Because previous studies have reported ventricular enlargement in MCI and AD (Thompson et al., 2004; Nestor et al., 2008) and in an rat model of GWI (Wu et al., 2021), in exploratory analyses we investigated effects of MCI, CDC CMI, and Kansas GWI group status (independent grouping variables) on hippocampal and lateral ventricular volumes (dependent variables). Imaging data from the SF 3T, SF MoCA, and GWIC cohorts were included in these exploratory analyses. Hippocampal and lateral ventricle volumes were entered into an omnibus multivariate analyses covariance (MANCOVA) with age, male sex, education, eTIV, PTSD, depression, and Kansas GWI exclusionary status as covariates. When there was significant main effect of group in the single omnibus MANCOVA, we used one-way ANCOVAs and pairwise comparisons with Bonferroni adjustments for multiple comparisons to further examine the effect of group on specific regional brain volume. Kansas GWI exclusionary status was not included as a covariate in the exploratory analysis of the effect of Kansas GWI status on regional brain volume.

We used Kruskal–Wallis and Chi-square tests to investigate group differences in deployment-related exposures because Shapiro–Wilk tests indicated that deployment-related exposure data was non-normally distributed.

All study procedures were approved by Institutional Review Boards (IRB) at the University of California, San Francisco, the SFVAHCS, Boston University, and the Department of Defense Office of Human and Animal Research Oversight. Informed consent was obtained from all GW veterans who participated in the original research studies at the SFVAHCS and who participated in the multi-site GWIC. GWIC study participants signed informed consents to share data for future studies.

## 3 Results

### 3.1 Study sample

Table 1 summarizes characteristics of the 5 GW veteran cohorts. The cohorts differed in age (*F*_4_,_951_ = 67.31, *p* < 0.001; all cohorts different from each other, *p* ≤ 0.003, except GWIC and SF 4T, *p* = 0.05), sex (χ^2^ = 9.73, df = 34 *p* < 0.05, SF MoCA cohort had fewest female veterans), years of formal education (*F*_4_,_951_ = 10.25, *p* < 0.001, SF 3T cohort > GWIC and SF 1.5T cohorts, *p* < 0.001), and race (χ^2^ = 106.76, df = 12, *p* < 0.001). There were fewer White veterans in the SF 1.5T and SF 4T cohorts. However, we did not collect information about race and ethnicity separately in the earlier SF cohorts. Therefore, veterans who self-identified as Hispanic were not asked if they also self-identified as White. Hispanic veterans from the SF 1.5T and SF 4T cohorts were categorized as “other” racially. The cohorts differed in ethnicity (χ^2^ = 17.58, df = 4, *p* < 0.001): the SF 4T cohort had the most veterans who self-identified as Hispanic.

The cohorts differed in rates of PTSD (χ^2^ = 48.15, df = 4, *p* < 0.001) and depression (χ^2^ = 91.73, df = 4, *p* < 0.001). The differences persisted even when we analyzed the data separately as a function of PTSD/depression assessment method (i.e., SCID/CAPS vs. self-report). There were differences in rates of depression (χ^2^ = 18.20, df = 1, *p* < 0.001 for self-report; χ^2^ = 58.92, df = 1, *p* < 0.001 for SCID) and PTSD (χ^2^ = 11.65, df = 1, *p* < 0.001 for self-report; χ^2^ = 5.88, df = 2, *p* = 0.05 for CAPS). There were fewer CMI cases in earlier SF cohorts (χ^2^ = 83.57, df = 4, *p* < 0.001), and more Kansas GWI cases in the GWIC cohort compared to the SF 3T and SF MoCA cohorts (χ^2^ = 99.07, df = 2, *p* < 0.001). However, we did not have the necessary information to ascertain Kansas GWI case status in the SF 1.5T and SF 4T cohorts. Rates of predicted Khamisiyah exposure differed in the SF 1.5T, SF 4T, and SF 3T cohorts (χ^2^ = 46.10, df = 2, *p* < 0.001). We did not have information about predicted Khamisiyah exposure for veterans in the GWIC or the SF MoCA cohorts.

### 3.2 Rates of MCI

**FIGURE 1:**
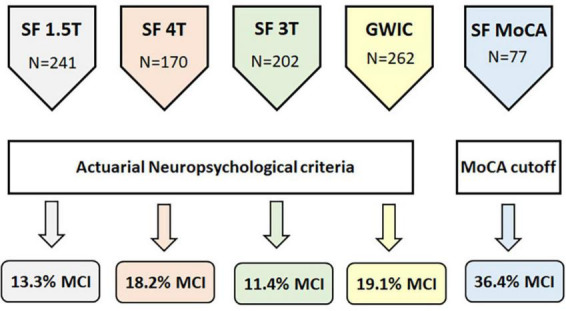
Schematic diagram showing the number of veterans in each cohort, how MCI status was ascertained, and the percent of veterans with MCI in each cohort.

The first aim of this study was to replicate our previous finding of a greater-than-expected (i.e., >10%) rate of MCI in additional cohorts of middle-aged GW veterans. We modified the actuarial MCI criteria used in our original study (Chao, 2020) to accommodate the neuropsychological tests that were available in the other cohorts. The actuarial MCI criteria employed in this study had two fewer measures and two fewer cognitive domains than the criteria that we used in our original study (Chao, 2020). As a result, the number of veterans classified as actuarial MCI cases in the SF 3T cohort was slightly, but not significantly, lower that previously reported (11.4% vs. 12.4%). The rates of actuarial MCI were marginally higher in the GWIC and SF 4T cohorts (χ^2^ = 7.04, df = 3, *p* = 0.07, see Figure 1).

In addition to the actuarial neuropsychological criteria, we also used the MoCA to determine MCI status in the SF MoCA cohort. Using a cut-off consistent with the *National Institute on Aging–Alzheimer’s Association’s* recommendation for identifying MCI (Albert et al., 2011), over a third of the veterans (36.4%) in the SF MoCA cohort were classified as MCI. The median age of the veterans with MCI was 60 years (range: 51–81 years). If we use the more liberal cut-off of MoCA <26, recommended by Nasreddine et al. (2005) to classify MCI, then more than half (57.1%) of the SF MoCA cohort would have been classified as MCI. The median age of veterans with MoCA scores <26 was also 60 years (range: 50–81 years).

The MCI, intermediate, and cognitively unimpaired groups differed on some demographic and clinical characteristics (see Table 3). There was a marginal effect of age (*F*_2_,_951_ = 3.01, *p* = 0.05); the intermediate group was older than the unimpaired group (Tukey’s *post-hoc*, *p* = 0.04). There was a significant effect of race (χ^2^ = 15.53, df = 2, *p* < 0.001); more unimpaired veterans self-identified as White compared to veterans in the MCI and intermediate groups. There was an effect of ethnicity (χ^2^ = 12.13, df = 2, *p* = 0.016); more veterans with MCI self-identified as Hispanic compared to veterans in the unimpaired and intermediate groups. There was an effect of CDC CMI status (χ^2^ = 13.40, df = 2, *p* = 0.001); more veterans classified as MCI also had CMI compared to the unimpaired and intermediate veterans. There was an effect of depression (χ^2^ = 12.19, df = 2, *p* = 0.004); more veterans with MCI had a history of depression compared to intermediate and unimpaired veterans. There was a marginal effect of PTSD (χ^2^ = 5.84, df = 2, *p* = 0.05); fewer unimpaired veterans had a history of PTSD compared to the MCI and intermediate veterans.

**TABLE 3:** Demographic and clinical characteristics by cognitive status.^1^

	Unimpaired	Intermediate	MCI	Statistics
*N* (%)	393 (41.3%)	395 (41.5%)	164 (17.2%)	
**Age, years**				
Mean (SD)	50.3 (8.6)	51.9 (9.5)	50.7 (8.8)	*p* = 0.05
Range	31.5–77.8	31–79.5	32–81.4	
Median	50.7	51.5	49	
**Education, years**				
Mean (SD)	14.9 (2.1)	15.0 (2.3)	15.0 (2.1)	n.s.
Range	10–20	11–21	12–21	
Median	14	14	15	
Male, *N* (%)	337 (86%)	333 (84%)	145 (88%)	n.s.
Ethnicity, *N* (%) Hispanic	33 (8%)	42 (11%)	29 (18%)	*p* < 0.02
Race, *N* (%) White	306 (79%)	266 (68%)	107 (65%)	*p* < 0.001
*N* (%) CDC CMI^a^	291 (74%)	295 (75%)	143 (88%)	*p* = 0.001
*N* (%) Kansas GWI	117 (67%)	135 (70%)	57 (75%)	n.s.
*N* (%) with Kansas GWI exclusionary condition(s)	33 (16%)	38 (16%)	24 (15%)	n.s.
*N* (%) with predicted Khamisiyah exposure	91 (34%)	75 (29%)	21 (24%)	n.s.
*N* (%) with history of PTSD^b^	124 (32%)	151 (39%)	66 (41%)	*p* = 0.05
*N* (%) with history of depression^c^	123 (32%)	137 (35%)	75 (47%)	*p* = 0.004

^1^Ascertained by actuarial neuropsychological method and Montreal Cognitive Assessment.

^*a*^Centers for Disease Control and Prevention Chronic Multisymptom Illness: fatigue, joint + muscle pain, difficulty sleeping, remembering, concentrating.

^*b*^Includes lifetime PTSD by CAPS and self-report.

^*c*^Includes DSM IV lifetime and current MDD and self-report.

We used multiple regression model to examine the relationship between demographic and clinical characteristics on MCI status. Because we combined data from the cohorts classified with the actuarial neuropsychological criteria and with the MoCA, we included the method of MCI classification as an independent variable. The overall model was significant (*R*^2^ = 0.06, *F*_9_,_929_ = 6.70, *p* < 0.001). Non-white race (β = 0.12, *p* < 0.001), MCI classification method (β = 0.15, *p* < 0.001), and history of depression (β = 0.09, *p* = 0.017) were a significant predictors of MCI status. Years of formal education (β = 0.07, *p* = 0.058), and Hispanic ethnicity (β = 0.06, *p* = 0.077) marginally predicted MCI status.

### 3.3 Regional brain volume differences as a function of MCI status

A second aim of the study was to replicate our previous finding of hippocampal atrophy in GW veterans with MCI. For this reason, we initially excluded hippocampal volume from the SF 3T cohort in the one-way ANCOVA that controlled for age, male sex, eTIV, years of education, history of PTSD and depression, and Kansas GWI exclusionary status (as a proxy for current health). There was a significant main effect of group (*F*_2_,_162_ = 3.14, *p* = 0.046), and pairwise comparisons revealed significant hippocampal volume differences between the unimpaired and MCI groups (*p* = 0.02) and marginal hippocampal volume differences between the unimpaired and intermediate groups (*p* = 0.06, see Table 4). There was also a significant group effect on group on hippocampal volume in the GWIC cohort alone (*F*_2_,_146_ = 3.26, *p* = 0.04). Planned contrasts revealed significant differences between the unimpaired and MCI groups (*p* = 0.02). There were not enough subjects in the SF MoCA cohort (MCI, four intermediate, five unimpaired) to analyze hippocampal separately in this cohort.

**TABLE 4:** Least square means^1^ hippocampal and lateral ventricle volumes^2^ by cognitive status.

	Unimpaired	Intermediate	MCI	Statistics		
				ANCOVA	Pairwise comparisons^3^	
	*n* = 67	*n* = 63	*n* = 33	*F*_2_,_162_	Unimp vs. MCI	Unimp vs. inter
Hippocampus^4^	8.65 (0.09)	8.41 (0.09)	8.30 (0.12)	3.14*	*	†
	*n* = 148	*n* = 156	*n* = 54	*F*_2_,_357_		
Hippocampus^4^	8.81 (0.05)	8.55 (0.05)	8.51 (0.09)	7.38***	**	**
Lateral ventricle^5^	19.57 (0.82)	23.40 (0.80)	21.73 (1.35)	5.49**		**

Unimp, unimpaired; inter, intermediate.

^1^Adjusted for age, male gender, years of education, eTIV, PTSD, depression, and Kansas GWI exclusionary status (as proxy for current health status) with standard error of the mean in parenthesis.

^2^Values (in cc) are left and right sides combined.

^3^Bonferroni adjustments for multiple comparisons.

^4^Excluding SF 3T cohort.

^5^In all subjects with 3T imaging data; main effect of group in single omnibus MANOVA: Wilks lambda = 0.90, Pillai’s approximate *F*_4_,_694_ = 4.90; *p* < 0.001.

*p < 0.05,

**p ≤ 0.01,

***p ≤ 0.001.

^†^*p* = 0.06.

Ventricular enlargement commonly co-occurs with hippocampal atrophy (Apostolova et al., 2012) and has been reported in patients with MCI and AD patients (Thompson et al., 2004; Nestor et al., 2008). Therefore, in *post hoc* analyses we examined the effects of cognitive status on hippocampal and lateral ventricle volume in the entire sample with 3T neuroimaging data (i.e., SF 3T, SF MoCA, and GWIC cohorts). The exploratory MANCOVA revealed a significant effect of group (Wilks lambda = 0.95, Pillai’s approximate *F*_4_,_694_ = 4.90, *p* < 0.001). Univariate tests revealed significant group differences in both hippocampal (*F*_2_,_357_ = 7.38, *p* < 0.001) and lateral ventricle (*F*_2_,_357_ = 5.49, *p* = 0.004) volumes. Kansas GWI exclusionary status, which was included as a proxy for current health, significantly adjusted the association between cognitive status and hippocampal volume (*p* = 0.026). Depression significantly adjusted the association between cognitive status and lateral ventricle volume (*p* = 0.038). Pairwise comparisons with Bonferroni adjustments for multiple comparisons revealed significant hippocampal volume differences between the unimpaired and MCI (*p* = 0.01) and intermediate (*p* = 0.002) groups. Pairwise comparisons revealed significant lateral volumes differences between the unimpaired and intermediate groups (*p* = 0.003, see Table 4).

### 3.4 Regional brain volume differences as a function of CDC CMI and Kansas GWI case status

Because Wu et al. (2021) reported hippocampal atrophy and lateral ventricle enlargement in a rat model of GWI, in exploratory analyses, we examined the effects of CDC CMI and Kansas GWI group status on hippocampal and lateral ventricle volumes. The single omnibus MANCOVA revealed no significant effect of CDC CMI case status (Wilks lambda = 1.0, Pillai’s approximate *F*_2_,_348_ = 0.24, *p* = 0.79) or Kansas GWI case status (Wilks lambda = 1.0, Pillai’s approximate *F*_2_,_294_ = 0.45, *p* = 0.64).

Previous neuroimaging studies GWI have reported subcortical (i.e., brainstem, thalamus, ventral diencephalon, and cerebellum) atrophy in veterans with GWI/CMI compared to control veterans (Christova et al., 2017; Zhang et al., 2020). Therefore, we also explored the effects of CDC CMI and Kansas GWI group status on the volumes of these subcortical regions. The single omnibus MANCOVA did not yield significant effects of CDC CMI case status (Wilks lambda = 1.0, Pillai’s approximate *F*_4_,_348_ = 0.13, *p* = 0.97) or Kansas GWI case status (Wilks lambda = 1.0, Pillai’s approximate *F*_4_,_294_ = 0.75, *p* = 0.56).

### 3.5 Relationship between GW-related exposures and cognitive status

**FIGURE 2:**
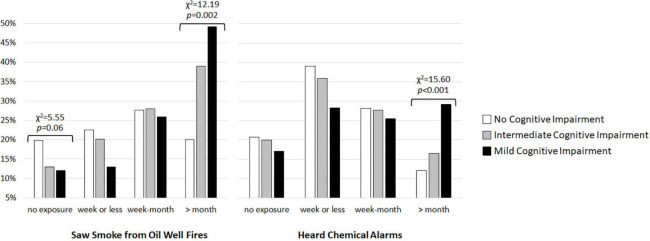
Percent of GW veterans with no cognitive impairment (white bars), intermediate cognitive impairment (gray bars), and mild cognitive impairment (MCI, black bars) who saw smoke from burning oil well filres and heard chemical alarms sound during deployment.

Kruskal–Wallis tests revealed significant group differences in exposure to oil well fire smoke (*H* = 14.62, df = 2, *p* < 0.001) and hearing chemical alarms sound (*H* = 8.60, df = 2, *p* = 0.01). There was a non-significant trend toward a group difference in exposure to pesticide fogging (*H* = 4.80, df = 2, *p* = 0.09). More veterans classified as MCI were exposed to smoke from oil well fires (χ^2^ = 12.19, df = 2, *p* = 0.002) and heard chemical alarms sound (χ^2^ = 15.60, df = 2, *p* < 0.001) for a month or longer during GW deployment compared to unimpaired and intermediate groups (see Figure 2).

## 4 Discussion

Cognitive dysfunction is common symptom among veterans with GWI/CMI (Smith et al., 2013; Janulewicz et al., 2017; Jeffrey et al., 2019). However, it had been unclear how severe the cognitive dysfunction associated with GWI/CMI is on the clinical spectrum. As a diagnostic entity, MCI has been proposed to describe individuals, primarily older adults, who have cognitive deficits greater than expected for their age, but who do not meet criteria for dementia (Petersen et al., 1999, 2009). This study replicated our previous finding of a higher than expected (>10%) rate of MCI in four additional cohorts of middle-aged GW veterans. The rate of MCI in the combined sample of 952 veterans was 17.2%; the median age of the veterans with MCI was 49 years old at the time of cognitive assessment. This is notable because research indicates that cognitive decline typically does not start to become apparent until individuals reach their 60s (Rabbitt et al., 2001; Barnes et al., 2007; Myers, 2008).

We used two methods to determine cognitive status in this study. Among veterans classified with the actuarial neuropsychological method, 15.5% met criteria for MCI (median age 50 at time of assessment). Among veterans classified with a MoCA cut-off score ≤23 previously validated in epidemiological settings (Luis et al., 2009), 36.4% of GW veterans met the criteria for MCI (median age 59 at time of assessment). How significant is a 16%–36% rate of MCI in a group of GW veterans who were 49–59 years old at the time of cognitive testing? It is double the estimated rate of MCI for individuals 60–64 years old reported in the American Academy of Neurology Practice update summary of MCI (Petersen et al., 2018). A recent review estimated that 11% of the population younger than 60 years old have MCI (Casagrande et al., 2022). Another review/meta-analysis estimated that 7.5% of adults between 55 and 59 have of MCI (Lu et al., 2021). If we use these estimates (6.7%, 7.5%, and 11%) as benchmarks, our finding that 16% of GW veterans met the actuarial criteria for MCI, and that 36% of GW veterans had MoCA scores indicative of MCI, is materially higher-than-expected.

The ideal group with which to compare rates of MCI would have been non-deployed GW-era veterans. Unfortunately, we did not have access to non-deployed GW-era who had been assessed with a comparable battery of neuropsychological tests. However, the Vietnam Era Twin Study of Aging (VETSA) was a study that assessed cognition in 51–59 year old men (Kremen et al., 2014). If we use the rate of MCI reported in the VETSA study as benchmarks (1.3% amnestic MCI and 6.0% non-amnestic-MCI), our finding that 16%–36% of GWVs 50–59 years old at the time of cognitive testing met criteria for MCI is also considerably higher-than-expected.

The subgroups of GW veterans with MCI, intermediate, and unimpaired cognitive status differed on some demographic and clinical characteristics. Consistent with reports that Black and Hispanic individuals have a greater likelihood of MCI compared to White individuals even after accounting for age and education differences (Wright et al., 2021), there were more Black and Hispanic veterans with MCI compared to White veterans. In line with previous reports linking depression with risk for developing cognitive impairment (Yaffe et al., 1999; Jorm, 2001; Ownby et al., 2006; Dotson et al., 2010; Barnes et al., 2012), more veterans with MCI had a history of depression compared to cognitively unimpaired and intermediate veterans. We also found marginally higher rates of PTSD among veterans with MCI and intermediate cognitive status compared to cognitively unimpaired veterans, consistent with reports of cognitive dysfunction in individuals with chronic PTSD (Golier et al., 2002; Yehuda et al., 2006).

Replicating our earlier finding (Chao, 2020), veterans with MCI had smaller hippocampal volume compared to veterans with unimpaired cognition in this study. Previous longitudinal studies have reported morphometric difference in the hippocampi of incident AD cases at least 10 years prior to diagnosis (den Heijer et al., 2010; Bernard et al., 2014; Coupe et al., 2017). In a study that compared two models (normal and pathological) of brain aging, Coupe et al. (2019) reported that the hippocampus was the first brain region to diverge from normal aging. Ventricle enlargement commonly co-occurs with hippocampal atrophy (Apostolova et al., 2012) and is frequently present in patients with MCI and AD (Thompson et al., 2004; Nestor et al., 2008). We found an effect of cognitive status on lateral ventricle volume; however, planned contrasts did not reveal significant differences between the cognitively unimpaired and MCI veterans (only differences between unimpaired and intermediate groups reached significance). Ventriculomegaly has been suggested to be an objective and sensitive measure of neuropathological change (Nestor et al., 2008). However, our data suggests that hippocampal volume may be more sensitive to neuropathological change in middle-aged GW veterans than lateral ventricular volume.

We found MCI to be more prevalent among veterans who met criteria for CDC CMI. This was not unexpected considering that cognitive dysfunction is one of the symptom categories for defining CDC CMI case status. However, another reason for this finding may relate to the suggestion that the CDC CMI case definition, developed 25 years ago, is increasingly capturing an overlapping amalgam of conditions associated 1990–1991 GW service and normal aging (Gifford et al., 2021). Consistent with this idea, the percentage of veterans who met CDC CMI case status increased as a function of the age of the GW veteran population: 65% among veterans (median age 46 years) assessed between 2002 and 2010; 84% among veterans (median age 51 years) assessed between 2014 and 2020; and 100% among veterans (median age 59 years) assessed between 2020 and 2023. Because the incidence of MCI increases with age (Ganguli et al., 2013), it is not surprising that it would be comorbid with another condition (CDC CMI) that is increasingly associated age.

In exploring the relationship between veterans’ cognitive status and deployment-related exposures, we found a significant relationship between two deployment-related exposures and MCI status: veterans with MCI reported longer exposures to smoke from oil well fires and hearing chemical sound compared to cognitively unimpaired and intermediate veterans. During Operation Desert Storm, Iraqi troops ignited 600 oil well fires (Department of Veterans Affairs, 2021) that produced dense clouds of soot, liquid, aerosols, and gases (World Meterological Organization [WMO], 1992). The combustion pollutants, estimated from the known composition of Kuwaiti crude oil likely included SO_2_, NO*_*x*_*, H_2_S, CO, suspended particulates, inorganic acids, metals, polycyclic aromatic hydrocarbons, and volatile organic compounds (Heller, 2011). Compared to cognitive unimpaired and intermediate veterans, veterans with MCI reported longer duration (≥31 days) exposure to smoke from oil well fires. This finding is in line with epidemiological studies that have reported associations between ambient pollution exposure (e.g., PM_2_._5_) and cognitive decline, all-cause dementia, and clinically diagnosed AD (Jayaraj et al., 2017; Peters et al., 2019; Russ et al., 2019; Tsai et al., 2019; Costa et al., 2020). Neuroinflammation (Block and Calderon-Garciduenas, 2009), oxidative stress (Costa et al., 2020), and microglia activation have been posed as a possible mechanisms by which air pollution causes neurotoxic effects in the brain (Block and Calderon-Garciduenas, 2009; Jayaraj et al., 2017).

Compared to veterans with unimpaired and intermediate cognition, veterans with MCI also reported a higher frequency of hearing chemical alarms sound for a month or longer during deployment. There were several incidents of chemical warfare agents release/exposure during the GW (United States Department of Defense, 1992; United States General Accountability Office, 1997; Tuite and Haley, 2013). The best-known incident occurred when a stockpile of enemy weapons containing nerve agents sarin and cyclosarin was destroyed at Khamisiyah, Iraq in March 1991 (Department of Defense [DoD], 2002). However, other exposures to chemical warfare agents, including those following Coalition bombings of Iraqi chemical facilities in the opening days of the war (Tuite and Haley, 2013) also occurred. Our finding of an association between the frequency of hearing chemical alarms sound and cognitive status is in line with animal (Filliat et al., 2007; Joosen et al., 2009; Mamczarz et al., 2011) and human (Pereira et al., 2014) studies that have reported cognitive deficits after exposure to chemical nerve agents and organophosphate chemicals. Similar to air pollutants, low levels of chemical warfare agents likely produce neurotoxic effects in the brain through neuroinflammation, oxidative stress, and microglia activation (O’Callaghan et al., 2017; Michalovicz et al., 2019).

This study has some limitations that warrant discussion. First, we relied upon review papers and meta-analyses of MCI prevalence studies to provide a comparison rate of MCI in adults under 60 years old. A better comparison group would have been non-deployed GW-era veterans of similar age. Second, although the Jak-Bondi actuarial neuropsychological diagnostic method is well-validated approach for studying MCI (Jak et al., 2009a,b, 2016; Clark et al., 2013; Bondi et al., 2014; Edmonds et al., 2016; Oltra-Cucarella et al., 2018; Wong et al., 2018; Graves et al., 2020, 2022; Fountain-Zaragoza et al., 2021; Devlin et al., 2022; Emmert et al., 2022), other methods have been used to diagnose MCI (Casagrande et al., 2022). Third, we did not verify MCI status with clinical neurological screenings. Because this study had a cross-sectional design, future longitudinal studies will be necessary to determine the prognosis of the GW veterans classified as MCI. Although not all studies agree on the impact of apolipoprotein (APOE) ε4 on MCI (Heun et al., 2010), MCI is commonly considered a prodrome for AD (Albert et al., 2011), and APOE ε4 is one of the strongest genetic risk factor for sporadic late-on-sent AD (Polsinelli et al., 2023). There have also been reports that APOE ε4 causes earlier age of symptom onset (Blacker et al., 1997; Bettens et al., 2013), faster ventricular expansion (Roussotte et al., 2014), and greater rates of hippocampal atrophy and cortical thinning in subjects with MCI (Abushakra et al., 2020). Therefore, another limitation of this study is our lack of information about APOE ε4 allele for all of the GW veterans in sample.

These limitations notwithstanding, the results from this and our previous study (Chao, 2020) suggest that GW veterans may not only be aging at a faster rate than the general population (Zundel et al., 2019), but may also be at increased risk for MCI and dementia. Because dementia has a long preclinical period (Jack et al., 2013), it may be prudent to council GW veterans, particularly those with CMI, history of depression, PTSD, and deployment-related exposures to oil well fire smoke and chemical nerve agents, to adopt lifestyle habits that have been shown to lower modifiable dementia risks (Zhang et al., 2021). The Food and Drug Administration (FDA) recently approved two anti-amyloid monoclonal antibodies for the treatment of AD and the Department of Veterans Affairs has decided to cover the cost for the lecanemab for veterans with MCI or mild AD. Because lecanemab is only indicated for patients with evidence of amyloid β (Aβ), a logical next step for this research is to determine if GW veterans with MCI have Aβ and tau in their brain and plasma.

## Data availability statement

The raw data supporting the conclusions of this article will be made available by the authors, without undue reservation.

## Ethics statement

The studies involving humans were approved by the Institutional Review Boards at University of California, San Francisco, Boston University, Miami VAMC, and Baylor College of Medicine and reviewed by the U.S. Army Medical Research and Development Command’s Office of Human Research Protections. The studies were conducted in accordance with the local legislation and institutional requirements. The participants provided their written informed consent to participate in this study. Additionally, GWIC participants signed informed consent to share data for future studies.

## Author contributions

LC: Conceptualization, Data curation, Formal analysis, Funding acquisition, Investigation, Writing – original draft, Writing – review & editing. KS: Funding acquisition, Writing – review & editing. MK: Writing – review & editing. RK: Data curation, Writing – original draft. LS: Writing – review & editing. NK: Writing – review & editing. B-BK: Writing – review & editing.
